# Moderate Nitrogen Application Synergistically Improved Yield and Quality of Nanjing Series *japonica* Rice Varieties with Good Taste

**DOI:** 10.3390/plants14060940

**Published:** 2025-03-17

**Authors:** Xiaodong Wei, Qingyong Zhao, Chunfang Zhao, Yong Zhang, Tao Chen, Zhen Zhu, Kai Lu, Lei He, Lihui Zhou, Shengdong Huang, Yusheng Li, Cailin Wang, Yadong Zhang

**Affiliations:** Institute of Food Crops, Jiangsu Academy of Agricultural Sciences, East China Branch of National Technology Innovation Center for Saline-Alkali Tolerant Rice, Nanjing Branch of China National Center for Rice Improvement, Jiangsu High Quality Rice Research and Development Center, Nanjing 210014, China; weiyinglin@163.com (X.W.); qingyong2001@163.com (Q.Z.); czhao@jaas.ac.cn (C.Z.); chentao19801014@126.com (T.C.); lukai@jaas.ac.cn (K.L.); helei@jaas.ac.cn (L.H.); zhoulihui@jaas.ac.cn (L.Z.); 19980010@jaas.ac.cn (S.H.);

**Keywords:** nitrogen level, *japonica* rice, high-quality cultivation, grain yield, good eating quality

## Abstract

Nanjing series *japonica* rice varieties developed by the Institute of Food Crops, Jiangsu Academy of Agricultural Sciences in China have the characteristics of an excellent taste quality, high yield, and good resistance. They are widely promoted and applied in the lower reaches of the Yangtze River in China’s *japonica* rice planting areas. In response to the problem of the lack of coordination between nitrogen fertilizer management measures and variety characteristics in production, which makes it difficult to synergistically improve yield and quality, this study adopted a split-plot experimental design to study the effect of nitrogen fertilizer application on yield and rice quality of Nanjing series *japonica* rice varieties. In 2021, four nitrogen application rates of 0 (N_1_), 150 (N_2_), 300 (N_3_), and 450 (N_4_) kg hm^−2^ (all pure nitrogen) were set up, and in 2022, four treatments of 120 (N_1_), 180 (N_2_), 240 (N_3_), and 300 (N_4_) kg hm^−2^ were set up, all with nitrogen application rate as the main plot factor and variety as the sub-plot factor. The results showed that the differences between the different nitrogen fertilizer treatments were significant at the 5% or 1% level, except for the milled rice rate, head rice rate, peak viscosity, setback viscosity, and paste temperature in 2021 and panicle number, grain number per panicle, all Rapid Visco-analyzer (RVA) characteristic values, and amylose content in 2022. With an increase in the nitrogen application rate, the number of panicles, grain number per panicle, and yield increased. Either the rates of brown rice, milled rice, or head rice and chalky grains or chalkiness showed an increase trend. The peak viscosity, hot viscosity, final viscosity, and breakdown viscosity decreased, while the setback viscosity increased. For the quality of cooked rice, the hardness increased, appearance, viscosity, and balance decreased, protein content increased, and taste value decreased. The interaction between nitrogen application rate and variety was significant at *p* < 0.05 or *p* < 0.01 only for yield components, processing quality, and rice protein content in 2021 and for eating and cooking quality, appearance quality, and peak viscosity in 2022. Other traits were not significant. The comprehensive results from two years of experiments showed that, under the conditions of this experiment, a nitrogen application rate of 240–300 kg hm^−2^ could improve the quality of rice in the Nanjing series varieties while maintaining a high yield. The results of this experiment have a guiding significance for the high-yield and high-quality cultivation of excellent-tasting Nanjing series *japonica* rice.

## 1. Introduction

Rice is the main grain crop in China, and Jiangsu Province is the main producer of rice in China, playing a crucial role in ensuring national food security. In the past 20 years, Jiangsu Academy of Agricultural Sciences and other institutions have made outstanding progress in the breeding of new varieties of excellent-tasting *japonica* rice, and have developed a number of *japonica* rice varieties with an excellent taste quality, high yield, and good resistance. Nanjing 46 (V1), Nanjing 9108 (V2), Nanjing 5718 (V3), Nanjing 9308 (V4), Nanjing 5818 (V5), Nanjing 5758 (V6), and Nanjingxiangnuo (V7) are all *japonica* rice varieties developed by the Institute of Food Crops, Jiangsu Academy of Agricultural Sciences [1,2,3,4,5]. Among them, V1–V5 are all semi-glutinous excellent-tasting *japonica* rice varieties containing the low amylose content gene *Wx^mp^*, with full growth periods of about 165 days, 153 days, 150 days, 147 days, and 145 days, respectively. The cooked rice is crystal clear, elastic, and does not harden after cooling, its taste is soft and smooth, and its taste quality is excellent. V6 is a late-maturing middle-season *japonica* rice variety with an excellent appearance and taste quality [5]. Its rice quality reaches the first grade of the agricultural industry standard “Cooking Rice Variety Quality (NY/T 593-2021)” [6]. V7 is an early-maturing late-season *japonica* glutinous rice variety, with a rice quality meeting the second level of the agricultural industry standard. Some of the above-mentioned varieties of Nanjing series *japonica* rice have just been promoted in production, while others have been promoted and planted in production for many years. The planting area of V1 exceeds 100,000 hectares, with a cumulative promotion area of over 800,000 hectares. V2 was promoted for a total of 4 million hectares from 2016 to 2024, with an annual planting area exceeding 350,000 hectares. It has become the largest rice variety in Jiangsu Province with the largest annual planting area for a single variety, and it was the third-largest variety of conventional rice promoted in China in 2023 [7]. V3 is a new variety of middle-season *japonica* rice with an excellent taste, strong lodging resistance, and high yield potential. Its promotion area exceeded 100,000 hectares by 2024 [3]. The planting area of V4 also exceeded 30,000 hectares in 2024.

During the planting process, it has been found that the yield and taste quality of the same variety of Nanjing series *japonica* rice planted in different years and locations, or by different farmers in the same year and location, vary greatly. Numerous studies have shown that nitrogen application rate has a significant impact on both rice yield and quality [8,9,10,11]. Li et al. (2007) [12] studied the effects of different nitrogen fertilizer application rates on the quality and yield of the rice variety Wuyujing 3. The results showed that with an increase in the nitrogen application rate in the range of 0–240 kg hm^−2^, the number of panicles, grains per panicle, yield, head rice rate, peck viscosity, and breakdown viscosity increased, while the chalky grain rate, chalkiness degree, and setback viscosity decreased. When the nitrogen application rate exceeded 240 kg hm^−2^, the seed setting rate, thousand-grain weight, yield, head rice rate, peck viscosity, and breakdown viscosity decreased, while the chalky grain rate, chalkiness degree, and setback viscosity increased. Based on this, it is believed that a nitrogen application rate of 240 kg hm^−2^ can be used as an indicator of nitrogen fertilizer application for a high yield and high quality of Wuyujing 3. Zhu et al. (2015) [13] studied the effects of different nitrogen fertilizer application rates on the yield and quality of the high-quality *japonica* rice Wuyunjing 27. The results showed that with an increase in the nitrogen application rate, the effective panicles, grain number per panicle, and yield first increased and then decreased, while the seed setting rate and thousand-grain weight showed a decreasing trend. The brown rice rate and milled rice rate first increased and then decreased, while the protein content of milled rice increased. The amylose content, taste value, and RVA spectrum characteristic value showed a decreasing trend. Based on the comprehensive yield and quality, the application rate of nitrogen fertilizer for Wuyunjing 27 should be controlled between 225 and 300 kg hm^−2^. Yu et al. (2016) [14] studied the effects of different nitrogen fertilizer application rates on yield and quality using three semi-glutinous *japonica* rice varieties as materials. The results showed that with an increase in the nitrogen application rate in the range of 0–450 kg hm^−2^, the yield showed an increasing trend, the chalky grain rate and chalkiness degree decreased, the brown rice rate, milled rice rate, and head rice rate increased, the gelatinization temperature increased, the peak viscosity decreased, the setback viscosity increased, and the taste quality deteriorated. The yield was the highest when treated with a nitrogen application rate of 300 kg hm^−2^. Zhu et al. (2017) [15] investigated the effects of nitrogen application rate on the grain yield and rice quality of two *japonica* soft super rice varieties, Nanjing 9108 and Nanjing 5055. The results showed that under seven nitrogen levels with application rates of 0, 150, 187.5, 225, 262.5, 300, and 337.5 kg hm^−2^, with an increasing nitrogen application level, the grain yields of both varieties first increased and then decreased. The highest yield was obtained at 300 kg hm^−2^. The milling quality and protein content increased, while the appearance quality, amylose content, gel consistency, cooking and eating quality, and rice flour viscosity decreased. They concluded that the suitable nitrogen application rate for the two varieties was 270 kg hm^−2^, under which they obtained a high yield and a superior eating and cooking quality. Chen et al. (2020) [16] studied the effects of different nitrogen application rates on the yield and quality of Nanjing 46. The results showed that increasing the amount of nitrogen fertilizer could delay the growth process of Nanjing 46, significantly increase plant height, leaf age, and effective panicle number, increase protein content, and reduce the taste quality of the rice. It was believed that the suitable nitrogen fertilizer application for Nanjing 46 was 240–285 kg hm^−2^, which balanced yield and taste. Liu et al. (2020) [17] studied the effects of different nitrogen fertilizer application rates on the yield and quality of the excellent-tasting *japonica* rice, Nanjing 9108, in the southern Henan region. The results showed that with an increase in the nitrogen application rate, the yield showed an increasing trend. The brown rice rate, milled rice rate, head rice rate, chalkiness, protein content, gel consistency, peak viscosity, and breakdown viscosity showed an increasing trend, while the chalky grain rate, amylose content, hot paste viscosity, final viscosity, setback viscosity, consistency viscosity, and gelatinization temperature showed a decreasing trend. The yield was highest in the treatment with a nitrogen application rate of 240 kg hm^−2^. In terms of taste quality, the appearance, viscosity, balance, and taste value of the cooked rice all showed a decreasing trend with an increase in the nitrogen application rate, while only hardness showed an increasing trend. Considering both yield and quality, a nitrogen application rate of around 240 kg hm^−2^ was beneficial for achieving a high quality and high yield of Nanjing 9108 in the southern Henan region. Wang et al. (2021) [18] studied the effect of nitrogen application rate on yield and quality using Jinchuan No.1 as their material. The results showed that increasing nitrogen fertilizer application could significantly improve the yield of Jinchuan No.1, but excessive nitrogen fertilizer application could lead to varying degrees of decrease in milling quality, processing quality, and taste quality. With an increase in nitrogen application, the protein and amylose content increased and the taste quality deteriorated significantly. Sun et al. (2019) [19] studied the effect of nitrogen application rate on the yield and quality of three Northeast japonica rice varieties. The results showed that yield increased with an increase in the nitrogen application rate, but the performance of taste quality was inconsistent among the three varieties. From this, it can be seen that most studies have shown that with an increase in the nitrogen application rate within the range of 0–300 kg hm^−2^, the number of panicles and yield increase. However, excessive nitrogen application can lead to a decrease in population quality, seed setting rate, yield, and nitrogen fertilizer utilization efficiency [20,21]. On the other hand, studies have also shown that with an increase in nitrogen application rate, the protein content of grains increases and the starch quality improves or decreases, but the taste quality decreases. Jiangsu is a high-yielding province, with the highest yield per hectare among the major rice-producing provinces in China and the highest nitrogen application rate, with some even exceeding 450 kg hm^−2^. In production practice, the excessive application of nitrogen fertilizer often leads to a decrease in rice quality in order to increase yield. In recent years, the quality of rice has received increasing attention. To improve rice quality, applying less nitrogen fertilizer in later stages has been emphasized, resulting in a decrease in yield. Due to the mismatch between nitrogen fertilizer application and variety characteristics, it is difficult to synergistically improve yield and quality. To ensure an excellent taste quality and yield of Nanjing series varieties and to achieve the matching of good varieties and good cultivation technologies, this study used the excellent-tasting Nanjing series *japonica* rice varieties as its materials to study the effect of the comprehensive regulation of nitrogen fertilizer application on their yield and quality, in order to provide a reference for the high-quality and efficient cultivation of Nanjing series varieties.

## 2. Materials and Methods

### 2.1. Experimental Treatment Design

The experiment was conducted at the Nanjing Base of Jiangsu Academy of Agricultural Sciences in 2021–2022. The soil was clay loam with 1.74% organic matter, 1.40 g kg^−1^ total nitrogen content, 32.1 mg/kg available phosphorus, and 165.8 mg kg^−1^ available potassium. According to previous research reports [12,13,14,15,16,17] and the actual fertilization situation in Jiangsu Province, the following four nitrogen application rates (all pure nitrogen) were set in 2021: 0 (N_1_), 150 (N_2_), 300 (N_3_), and 450 kg hm^−2^ (N_4_). According to the experimental results of 2021, the following four treatments were set for nitrogen fertilizer treatment in 2022: 120 (N_1_), 180 (N_2_), 240 (N_3_), and 300 kg hm^−2^ (N_4_). For both years, a split-plot design was adopted, with nitrogen application rate as the main plot factor and variety as the sub-plot factor. The main plot and sub-plot were randomly arranged and repeated three times. Soil embankments were built between each residential area for isolation and separate irrigation and drainage. In total, 18 rows were planted in each plot, with 18 plants per row and 3 seedlings per hole, with a row spacing of 25 cm x 13.3 cm. On May 10th, the seeds were soaked in liquid medicine and sown on May 14th, using hard-ground seedling trays and the sprinkler irrigation method to raise seedlings. The seedlings were transplanted on June 10th. Except for the N_1_ treatment in 2021, each plot uniformly used compound fertilizer with a N-P_2_O_5_-K_2_O content of 20%, 12% and 16% as the base fertilizer, with rotary tillage performed on the soil during land preparation. After transplanting 7 and 14 days later, urea was applied as a tillering fertilizer. When the fourth to last leaf came out, an equal amount of compound fertilizer with a N-P_2_O_5_-K_2_O content of 16%, 0% and 16% was used as a panicle fertilizer for each treatment, while urea was used for the remaining nitrogen fertilizer to ensure the same amount of phosphorus and potassium fertilizers for each treatment. The nitrogen fertilizer management mode was base fertilizer–tiller fertilizer–panicle fertilizer = 4:4:2. When the total number of tillers reached around 270 × 10^4^ hm^−2^, the fields were drained. After finishing the drainage, except for establishing a shallow water layer from the booting stage to the flowering stage, the rest of the time alternated between dry and wet irrigation, with moist irrigation. That was, after watering once in shallow water, we let it naturally fall dry, and then watered again in shallow water until reaching maturity. No more watering was applied from 10 days before harvest. Other cultivation management measures were implemented according to conventional cultivation requirements.

### 2.2. Test Varieties

In 2021, the tested rice varieties were Nanjing 46 (V1), Nanjing 9108 (V2), Nanjing 9308 (V4), and Nanjing Xiangnuo (V7). In 2022, Nanjing 9108 (V2), Nanjing 5718 (V3), Nanjing 5818 (V5), and Nanjing 5758 (V6) with relatively consistent growth stages were used. Except for V6, which is a non-semi-glutinous rice, and V7, which is a glutinous rice, all other varieties contain the low amylose content gene *Wx^mp^* and belong to the semi-glutinous good-tasting *japonica* rice varieties. The tested varieties have been widely promoted and planted in Jiangsu Province and its surrounding provinces.

### 2.3. Characteristic Determination and Methods

#### 2.3.1. Determination of Yield Traits

On the second day after transplantation, starting from the third hole in the diagonal second row of each plot, there were 5 consecutive holes, with 3 seedlings designated for each hole. Any excess 3 seedlings would be removed, and any less than 3 seedlings would be replenished. After maturity, these 10 holes were harvested and brought back indoors. The number of panicles in each hole were investigated, threshed separately, packed in sandbags, and left to dry. When the moisture content was around 14%, we measured the total number of grains, empty grains, and thousand-grain weight in each hole, calculated the number of grains per panicle and the setting rate, and took the average of the 10 holes as the characteristic value of the plot. All other plants in each plot were harvested, threshed, cleaned, and dried to a moisture content of about 14% to determine the yield of the plot, which was converted into yield per hectare.

#### 2.3.2. Determination of Processing Quality

A total of 10 g (W_1_) of rice that had been tested for thousand-grain weight was randomly weighed using a small brown rice mill (JLGJ4.5 model, Taizhou Grain Instrument Factory, Taizhou, China) to remove the rice husk, and then weighed (W_2_) to calculate the brown rice rate (=W_2_/W_1_, %). A small rice milling machine (BLH-3120, Zhejiang Bethlehem Instrument Equipment Co., Ltd., Taizhou, China) was used to grind and weigh brown rice (W_3_), and the rice milling rate was calculated (=W_3_/W_1_, %). After removing the broken rice from the milled rice, it was weighed (W_4_) and the head rice rate was calculated (=W_4_/W_1_, %). The measurement was repeated three times in each plot, and the average was taken as the plot trait value.

#### 2.3.3. Determination of Protein Content

A total of 10 g of milled rice was weighed and an experimental mill (JFS-13, Zhejiang Hangzhou Qianjiang Instrument Equipment Co., Ltd., Hangzhou, China) was used to grind it into rice powders. After passing through a 100 mesh sieve, it was put into a paper bag for standby. The total nitrogen content in the rice powders was determined by Kjeltec 8400 (FOSS, Hillerød, Denmark) and then multiplied by the conversion coefficient 5.95 to calculate the protein content (PC) of the rice powders. The measurement was repeated three times and the average was taken as the plot trait value.

#### 2.3.4. Determination of RVA Characteristic Values

The viscosity of the rice powders was measured by an RVA viscosity tester (TechMaster, Perten, Stockholm, Sweden), and the parameters were set according to the operating procedures of AACC61-01 and 61-02 of the American Grains Chemical Association. In total, 3 g of rice powders was put into an aluminum box and then mixed with 25 mL of distilled water. First, we used a plastic paddle with a speed of 960 rpm to stir the sample in the instrument pool, and then reduced the speed to 160 rpm. The sample was heated from 50 °C to 95 °C, then cooled back to 50 °C. Peak viscosity (PV), hot viscosity (HV), final viscosity (FV), pasting temperature (PaT), and peak time (PeT) were measured. The breakdown viscosity (BDV = PV − HV) and the setback viscosity (SBV = FV − PV) were calculated. The measurement was repeated for each sample three times and the average value was taken.

#### 2.3.5. Determination of Rice Taste Index

The appearance, hardness, viscosity, balance, and taste value of cooked rice were measured using a rice taste meter (STA-1A, Satake Company, Hiroshima, Japan). In 2022, the chalky grain rate, chalkiness degree, amylose content, and gel consistency were also measured.

#### 2.3.6. Determination of Nitrogen Fertilizer Utilization Efficiency

The agronomic nitrogen use efficiency (ANUE) and partial factor productivity of nitrogen (PFPN) were measured according to the following formula [22].

ANUE (kg kg^−1^) = (yield in nitrogen application area−yield in nitrogen free area)/nitrogen application rate (1)

PFPN (kg kg^−1^) = yield of nitrogen application area/nitrogen application rate (2)

### 2.4. Data Analysis

According to the method introduced by Mo [23], variance analysis was performed in Excel using a self-developed program, and Duncan’s new complex extreme difference method was used for multiple comparisons. Drawing was conducted in Excel.

## 3. Results and Analysis

**Figure 1:**
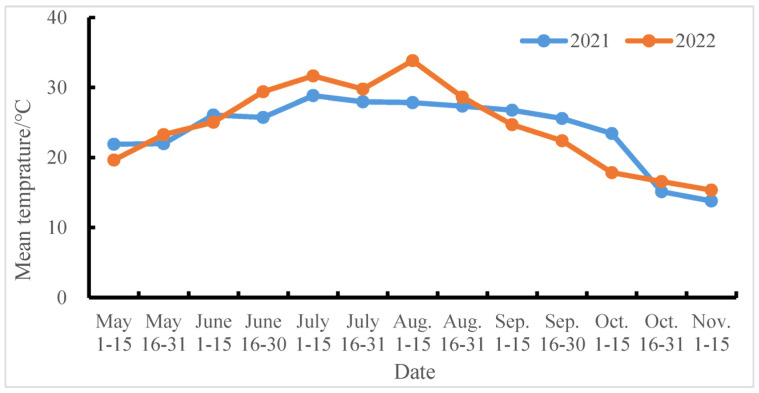
Average temperature during the rice growth period in the experimental area from 2021 to 2022.

According to the meteorological data provided by the Nanjing Meteorological Observatory, the temperature, rainfall, and light during the rice growing period in 2021 were normal annual conditions close to the annual average. Furthermore, 2022 was a year of high temperature, with 33 out of 45 days from July 10th to August 23rd having the highest temperature above 35 °C (Figure 1), which affected the setting rate of the experimental materials. Therefore, the yield of the tested varieties in 2022 was relatively low.

Variance analysis of the random split-block design was conducted on two years of data, and the results showed that, in 2021, the main plot errors of all traits did not reach a significance level of 5%. In 2022, except for the hardness and balance of cooked rice, chalky grain rate, chalkiness degree, and yield, the main plot errors of other traits also did not reach a significance level of 5%. Therefore, for traits with insignificant main plot errors, the main plot errors and split-plot errors were combined and retested. The results showed that the differences between different nitrogen application rates were significant at the 1% level, except for the milled rice rate, head rice rate, PV, SBV, and PaT in 2021 and the panicle number, grain number per panicle, all RVA characteristic values, and amylose content in 2022. The differences between varieties were significant at the 5% or 1% level, except for the number of grains per panicle and hardness of cooked rice in 2021 and the number of grains per panicle, seed setting rate, grain weight per plant, brown rice rate, milled rice rate, and PaT in 2022. The interaction between nitrogen application rate and variety reached a significance level of 5% or 1% only in terms of yield factors, processing quality, and rice protein content in 2021 and only in terms of eating and cooking quality, appearance quality, and peak viscosity in 2022 (Table 1, Table 2, Table 3 and Table 4).

### 3.1. The Effect of Nitrogen Application Rate on the Yield and Yield Components of Nanjing Series japonica Rice with Good Taste

**Figure 2:**
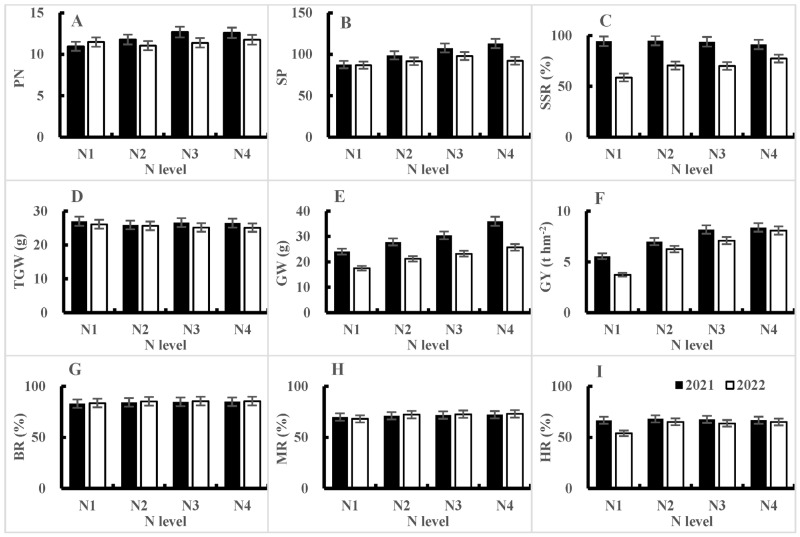
Response of yield traits and processing quality to nitrogen application levels in 2021 and 2022. PN = panicle number per plant, SP = spikelets per panicle, SSR = seed setting rate, TGW = 1000-grain weight, GW = grain weight per plant, GY = grain yield, BR = brown rice percentage, MR = milled rice percentage, and HR = head rice percentage.

From the average values of different nitrogen fertilizer treatments, it can be seen that, in 2021, with an increase in the nitrogen application rate, the number of panicles and grains per panicle increased. The seed setting rate first rose and then fell, showing an overall downward trend. The thousand-grain weight also showed a decreasing trend, while the grain weight per plant and yield increased. The N_3_ treatment had the highest number of panicles, with no significant difference compared to the N_4_ treatment, but significant differences compared to the N_2_ and N_1_ treatments. The N_4_ treatment had the highest number of grains per panicle, with no significant difference compared to the N_3_ treatment, but significant differences compared to the N_2_ and N_1_ treatments. The seed setting rate of the N_2_ treatment was the highest, with no significant difference compared to the N_1_ and N_3_ treatments, but a significant difference compared to the N_4_ treatment. The N_1_ treatment had the highest thousand-grain weight, but except for a significant difference compared to the N_2_ treatment, there was no significant difference compared to the N_3_ and N_4_ treatments. The yield was the highest in the N_4_ treatment, with no significant difference compared to the N_3_ treatment, but significant differences compared to the N_2_ and N_1_ treatments (Table 5, Figure 2A–F).

**Figure 3:**
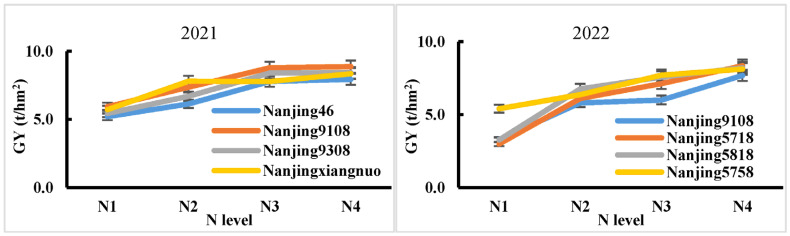
Yield responses of different varieties to nitrogen application levels.

The differences in the number of panicles and grains per panicle between different nitrogen fertilizer levels in 2022 were not significant, but showed an increasing trend. With an increase in the nitrogen application rate, the seed setting rate showed a significant increasing trend, with the N_1_ treatment being the lowest, the N_4_ treatment being the highest, and the N_2_ and N_3_ treatments being in the middle. The difference between N_2_ and N_3_ was not significant, but they were significantly different from N_1_ and N_4_ (Table 6, Figure 2A–F). This was opposite to the trend of change in 2021, as the abnormally high temperatures in July and August 2022 led to a significant decrease in the seed setting rate of the tested varieties. As the nitrogen application rate increased, the thousand-grain weight decreased, while the grain weight per plant and plot yield increased, which was consistent with the trend in 2021. Moreover, there was no interaction between nitrogen application rates and varieties in terms of yield over the two years, indicating that the yield response trends of the tested varieties were consistent under different nitrogen application rates (Figure 3).

From the results of two years of experiments, it can be seen that the treatment with the highest nitrogen application rate (N_4_) had the highest yield and the most grains per panicle, but differences from the N_3_ treatment were not significant. The N_3_ (2021) and N_4_ (2022) treatments had the highest number of panicles, and the difference between the N_3_ and N_4_ treatment was not significant over the two years.

**Figure 4:**
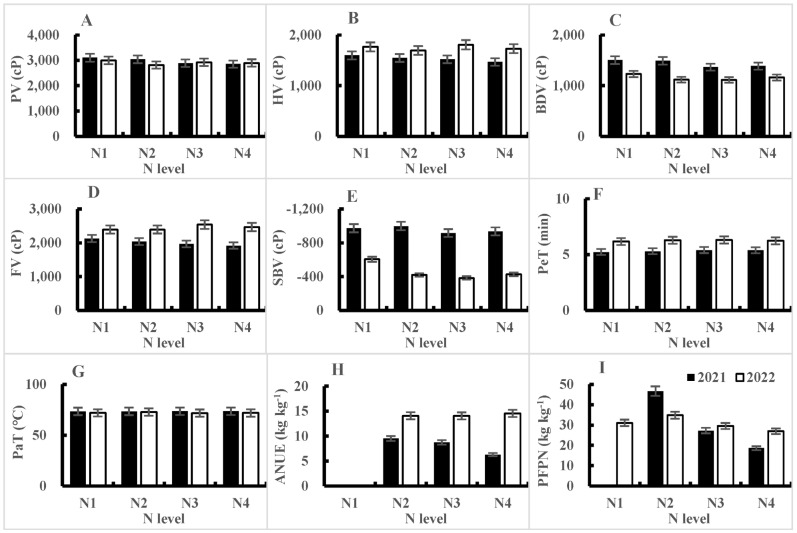
Response of RVA characteristics and N use efficiency to nitrogen application levels in 2021 and 2022. PV = peak viscosity, HV = hot viscosity, BDV = breakdown viscosity, FV = final viscosity, SBV = setback viscosity, PeT = peak time, PaT = pasting temperature, ANUE = agronomic nitrogen use efficiency, and PFPN = partial factor productivity of nitrogen.

**Figure 5:**
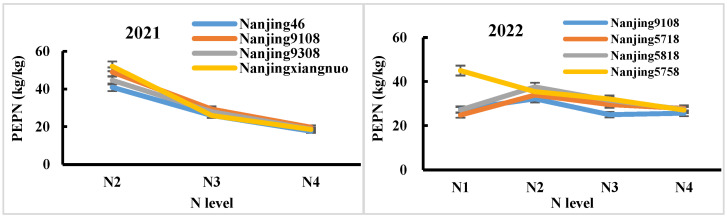
Partial factor productivity of nitrogen (PEPN) for different varieties at different nitrogen application levels.

From the perspective of nitrogen fertilizer utilization efficiency, both the agronomic nitrogen use efficiency (ANUE) and partial factor productivity of nitrogen (PFPN) decreased with an increase in the nitrogen application rate, and this trend was consistent over the two years (Table 5 and Table 6, Figure 4H,I). The results of the analysis of variance showed that the ANUE only showed significant differences between nitrogen application rates in 2021 and between varieties in 2022. However, the differences in nitrogen application rates, varieties, and their interactions for PFPN were all significant at the 1% level in both years. From Figure 5, it can be seen that, in 2021, there were significant differences in PFPN among varieties at low nitrogen levels (N_2_), with the largest being Nanjingxiangnuo, followed by Nanjing 9108. As the nitrogen application rate increased, the differences between varieties gradually decreased. In 2022, Nanjing 5758 had the highest PFPN under a low nitrogen level (N_1_), far exceeding the other three varieties, all of which had the highest PFPN under the N_2_ treatment.

### 3.2. The Effect of Nitrogen Application Rate on the Quality of Nanjing Series japonica Rice with Good Taste

#### 3.2.1. Impact on Processing and Appearance Quality

From the average values of processing quality under different nitrogen fertilizer treatments, it can be seen that with an increase in the nitrogen application rate, the rates of brown rice, milled rice, and head rice all showed an upward trend. Except for the head rice rate, which first showed an increase and then decrease (2021) or some increase and decrease with little change (2022), the N_1_ treatment had the lowest rate, which was significantly different from the other three treatments, while the differences between the other three treatments were not significant (Table 5 and Table 6, Figure 2G–I). The interaction between the nitrogen application rate and variety showed that, in 2021, the brown rice rate, milled rice rate, and head rice rate all reached a significance level of 5% or 1%. However, in 2022, the three traits were not significant, because the differences in the brown rice rate and milled rice rate among the four varieties tested in 2022 were not significant (Table 1 and Table 2, Figure 2G–I). The chalky grain rate and chalkiness degree were only detected in 2022, and their trend of change was similar to that of processing quality. The N_1_ treatment had the lowest chalkiness rate, and with an increase in the nitrogen application rate, the chalkiness rate and chalkiness degree increased. The changes between the N_2_, N_3_, and N_4_ treatments were not significant (Table 6). The interaction between the nitrogen application rate and variety, as well as the chalky grain rate and chalkiness degree, reached a significance level of 1% (Table 2), indicating that, with an increase in the nitrogen application rate, the change trends of the chalky grain rate and chalkiness degree in different varieties were significantly different.

#### 3.2.2. Impact on Eating and Cooking Quality

**Figure 6:**
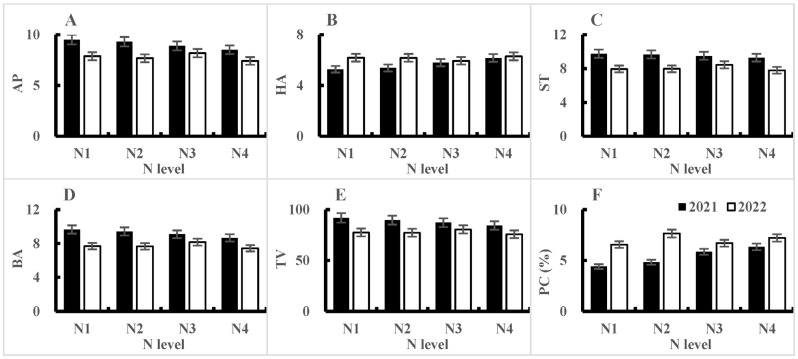
Response of eating and cooking quality traits to nitrogen application levels in 2021 and 2022. AP = appearance of cooked rice, HA = hardness of cooked rice, ST = stickiness of cooked rice, BA = balance of cooked rice, TV = taste value, and PC = protein content.

The response trends of the eating and cooking quality traits of the tested varieties in 2021 were consistent under different nitrogen application rates. The interaction between varieties and nitrogen application rates, except for protein content reaching a significance level of 1%, did not reach a significance level of 5% in terms of the appearance, hardness, viscosity, balance, and taste value of cooked rice (Table 3). From the average values of the tested varieties, the trend of change in eating and cooking quality was that as the nitrogen application rate increased, the protein content increased, the appearance, stickiness, and balance of cooked rice decreased, the hardness of cooked rice increased, and the taste value decreased. From the significance of the differences in mean values, the protein content and hardness of cooked rice were highest in the N_4_ treatment, with no significant difference compared to the N_3_ treatment, but with significant differences compared to the N_2_ and N_1_ treatments. The appearance, stickiness, and balance of cooked rice were the highest in the N_1_ treatment, but except for significant differences from the N_4_ treatment, there were no significant differences compared to the N_2_ and N_3_ treatments. The taste value of the N_1_ treatment was also the highest, with no significant difference compared to the N_2_ treatment, but significant differences compared to the N_3_ and N_4_ treatments. The differences between the N_3_ and N_4_ treatments also reached a significant level (Table 7, Figure 6).

**Figure 7:**
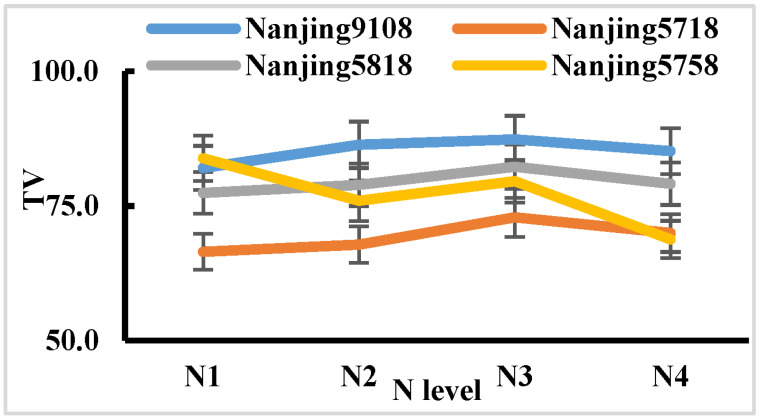
Taste values (TVs) for different varieties under different nitrogen application levels in 2022.

The response trends of the eating and cooking quality traits of the tested varieties in 2022 were inconsistent under different nitrogen application rates, and there was a highly significant interaction between nitrogen application rates and varieties (Table 4). The taste values of V2, V3, and V5 showed a downward opening parabolic relationship with nitrogen application rate, with the N_3_ treatment having the highest taste value. The taste value of V6 was highest in the N_1_ treatment, and gradually decreased with an increase in the nitrogen application rate (Figure 7). From the average values of the tested varieties, the changes in eating and cooking quality traits under different nitrogen application rates were not so large as in 2021. The hardness of cooked rice was the lowest in the N_3_ treatment, while the appearance, viscosity, balance, and taste values of cooked rice were the highest in the N_3_ treatment. The protein content was the lowest in the N_1_ treatment and lower in the N_3_ treatment. It can be seen that, for most of the eating and cooking quality traits, the N_3_ treatment was best (Table 8, Figure 6).

#### 3.2.3. Impact on RVA Characteristics

The analysis of variance of RVA characteristics showed that there were no significant differences among all the traits among the different nitrogen fertilizer treatments in 2022. In 2021, except for insignificant differences in the HV, SBV, and PaT among the different nitrogen fertilizer treatments, all other traits reached a significance level of 5% or 1%. The interaction between nitrogen fertilizer treatment and variety did not reach a significant level for all the traits over the two years (Table 3 and Table 4).

From the average RVA characteristic values of the different nitrogen fertilizer treatments in 2021, it can be seen that with an increase in the nitrogen application rate, the PV, BDV, and FV all showed a downward trend, while the PeT showed an upward trend. The changes in HV, SBV, and PaT were not significant (Table 7, Figure 4A–G). From the significance of the differences in mean values, the PV and BDV were highest in the N_1_ treatment and lowest in the N_4_ treatment. There was no significant difference between the N_1_ and N_2_ treatments, or between the N_4_ and N_3_ treatments. The FV was also highest in the N_1_ treatment and lowest in the N_4_ treatment, but except for significant differences between the N_1_ and N_3_ treatments and N_1_ and N_4_ treatments, the differences between the other treatments were not significant. The PeT of the N_1_ treatment was the shortest, with significant differences compared to the N_2_, N_3_, and N_4_ treatments, while the differences between the N_2_, N_3_, and N_4_ treatments were not significant (Table 7, Figure 4A–G).

### 3.3. Correlation Analysis Between Different Characteristics

The correlation analysis (Table 9) showed that, among yield traits, the yield in 2021 was significantly and extremely significantly positively correlated with the number of panicles per plant and grains per panicle, respectively. The grain weight per plant was highly significantly positively correlated with the panicle number per plant and grains per panicle. In 2022, yield was significantly positively correlated with the seed setting rate and grain weight per plant, while the number of panicles was significantly negatively correlated with the thousand-grain weight and the grain weight per plant was significantly positively correlated with the seed setting rate. The correlations between other yield traits were not significant. The processing quality traits of the brown rice rate, milled rice rate, and head rice rate were significantly positively correlated with each other for two years. There was a significant correlation among the six taste quality traits, with taste values showing a significant negative correlation with the protein content and hardness of cooked rice, and a significant positive correlation with the rice appearance, stickiness, and balance of cooked rice. Among the RVA characteristic values, most traits were significantly correlated with each other. PV was significantly positively correlated with BDV, FV was significantly correlated with HV and SBV, and PeT was significantly positively correlated with HV, FV, and SBV over the two years.

From the correlations between different types of traits (Table 9), although there was a significant correlation between the yield traits, eating and cooking quality, and RVA characteristic values for most traits, the performance of most correlations was inconsistent over the two years, indicating that the correlation between these traits was unstable and greatly influenced by environmental factors such as climate and cultivation. This could have been due to the significant impact of the abnormal high temperature in 2022 on the seed setting rate and yield, while 2021 was a normal year. Therefore, from the correlation between eating and cooking quality traits and yield traits in 2021, the taste value, appearance, viscosity, and balance of cooked rice were significantly or extremely significantly negatively correlated with yield, grain weight per plant, number of panicles, and number of grains per panicle. The hardness of cooked rice was significantly or extremely significantly positively correlated with yield, grain weight per plant, number of panicles, and number of grains per panicle. The yield and grain weight per plant were significantly positively correlated with the brown rice rate for two years, while taste value was highly significantly positively correlated with the appearance, hardness, stickiness, and balance of cooked rice over the two years, and significantly negatively correlated with protein content. In addition, according to the measurement results in 2022, the chalky grain rate and chalkiness degree were significantly positively correlated with the head rice rate, BDV, and PaT and significantly negatively correlated with SBV. The amylose content was significantly positively correlated with HV, FV, SBV, and PeT and significantly negatively correlated with BDV.

## 4. Discussions

### 4.1. Increasing Nitrogen Fertilizer Application Could Improve Yield with an Increase in the Number of Panicles and Grains per Panicle of Good-Eating-Quality japonica Rice

Nitrogen is the most important nutrient element for rice growth, which has a significant impact on rice yield and quality formation [24,25,26]. Nitrogen is also a component of various enzymes in rice plants, and enzymes are essential biocatalysts in the plant growth process. Nucleic acids, nuclear proteins, alkaloids, vitamins, and hormones also contain nitrogen [27], indicating that nitrogen plays a very important role in the growth and development of rice. Therefore, increasing nitrogen fertilizer application has become an important measure to increase yield in rice cultivation. However, more nitrogen fertilizer is not necessarily better. Numerous studies have shown that the response curve of rice yield to nitrogen fertilizer application follows a parabolic relationship with an opening downward trend [28]. Under an appropriate range of nitrogen fertilizer application and reasonable nitrogen fertilizer management, rice yield increases with an increase in the nitrogen application rate. However, once nitrogen fertilizer exceeds the appropriate amount or the management method is unreasonable, this can actually cause the excessive growth of rice, reduce lodging resistance and stress resistance, and decrease yield [29]. The results of this study indicated that with an increase in the nitrogen application rate, the number of panicles increased, the number of grains per panicle increased, and the overall grain setting rate showed a decreasing trend (except for the influence of a high temperature in 2022). The thousand-grain weight also showed a decreasing trend, and the yield increased with an increase in the nitrogen application rate (Table 3 and Table 4). There were significant differences in the response of yield factors to nitrogen application rate among varieties in 2021 (the interaction between nitrogen application rate and varieties was significant) (Table 1). Except for Nanjingxiangnuo with the highest number of panicles in the N_3_ treatment, Nanjing 46, Nanjing 9108, and Nanjing 9308 all had the highest numbers of panicles under the N_2_ treatment. The number of grains per panicle for Nanjing 9108 and Nanjing 9308 was also the highest in the N_2_ treatment. The seed setting rate of Nanjing 9108 in the N_2_ treatment was the highest, while Nanjing 46, Nanjing 9308, and Nanjingxiangnuo were all the highest in the N_1_ treatment. The correlation analysis showed that the number of panicles and grains per panicle were significantly positively correlated with yield (Table 9), indicating that with an increase in the nitrogen application rate, although the seed setting rate and thousand-grain weight of the Nanjing series *japonica* rice varieties showed a downward trend, the significant increase in the number of panicles and grains per panicle ultimately led to an increase in yield. The response of the yield factors to nitrogen application rate in 2022 showed a consistent performance among varieties (the interaction between nitrogen application rate and varieties was not significant).

From this, it can be seen that, although the N_4_ treatment with a nitrogen application rate of 450 kg hm^−2^ had the highest yield in 2021, there was no significant difference compared to the N_3_ treatment with a nitrogen application rate of 300 kg hm^−2^. The N_3_ treatment had the highest number of panicles, and there was no significant difference in the number of grains per panicle, seed setting rate, and thousand-grain weight between the N_3_ treatment and the treatment with the highest values. The N_4_ treatment with a nitrogen application rate of 300 kg hm^−2^ in 2022 had the highest yield, the most panicles, the highest seed setting rate, and the highest grain weight per plant. Although the thousand-grain weight was the lowest, the differences between treatments were small and the differences in grain number per panicle between treatments were not significant. Therefore, based on the results over two years, a nitrogen application rate of 300 kg hm^−2^ was the optimal treatment for the yield and yield components of the tested varieties.

### 4.2. Excessive Nitrogen Fertilizer Application Could Lead to Poor Gelatinization Characteristics, Increased Protein Content, and Decreased Taste Quality of Good-Eating-Quality japonica Rice

There have been many research reports on the relationship between nitrogen nutrition and quality [30,31,32,33,34]. The studies by Xu et al. (2004) [35] and Liu et al. (2004) [36] showed that, as the nitrogen application rate increased, the SBV gradually increased, while the PV, HV, FV, and BDV gradually decreased. Li et al. (2012) [37] found that with an increase in the nitrogen fertilizer level, the SBV and PaT gradually increased, while the changes in HV, FV, and consistency viscosity showed no obvious pattern. Jin et al. (2004) [38] found that with an increase in nitrogen application rate, the PaT tended to increase, but there was no significant difference between a treatment applying 5 g of nitrogen per pot and a treatment without fertilization, which was consistent with the research conclusions of Jin et al. (2001) [39] and Han et al. (1997) [40]. Kou et al. (2003) [41] and Yang et al. (2002) [42] believed that the amount of nitrogen fertilizer used had no significant effect on the PaT. The results of this study indicated that, as the nitrogen application rate increased, the brown rice rate increased, while the milled rice rate and head rice rate did not change significantly. The protein content increased, while the appearance, viscosity, and balance of cooked rice decreased, the hardness of cooked rice increased, and taste value decreased (Table 5, Table 6, Table 7 and Table 8). The PV, BDV, and FV all showed a decreasing trend, while the PeT showed an increasing trend. There were no significant changes in the HV, BDV, and PaT (Table 3 and Table 4). Except for the brown rice rate, milled rice rate, head rice rate, and protein content in 2021 and the rice texture traits, taste value, protein content, PV, appearance quality, and gel consistency in 2022, the response trends of other quality traits and RVA values to the nitrogen application rate were consistent among varieties (the interaction between nitrogen application rate and varieties was not significant) (Table 3 and Table 4). Correlation analysis showed that taste value was significantly negatively correlated with rice hardness and protein content and significantly positively correlated with rice appearance, viscosity, and balance (Table 9). This indicated that with an increase in nitrogen application rate, the appearance, viscosity, and balance of cooked rice in the Nanjing series varieties decreased due to the increase in protein content, while hardness increased, ultimately leading to a decrease in taste value.

### 4.3. Moderate Nitrogen Fertilizer Application Could Synergistically Improve the Yield and Quality of Good-Eating-Quality japonica Rice

It is generally believed that a high yield and high quality of rice are contradictory, and a high yield often leads to a decline in quality. The negative correlation between taste value and yield (r = −0.690) and the significant negative correlation between taste value and protein content (r = −0.724) in previous studies and the related analysis results in this study all prove this point. Yield increases with an increase in nitrogen application rate, while taste value decreases with an increase in nitrogen application rate. But, as mentioned earlier, yield will not increase infinitely with an increase in nitrogen application, and the same goes for quality. Wei et al. (2012) [43] studied the high-quality *japonica* rice Nanjing 46 and set seven nitrogen application levels, including 0, 150, 187.5, 225.0, 262.5, 300, and 337.5 kg hm^−2^. They found that, as the nitrogen application rate increased, the brown rice rate, milled rice rate, and head rice rate of Nanjing 46 showed an increasing trend. However, when the nitrogen application rate reached 262.5 kg hm^−2^, there was no significant difference compared to the treatments with nitrogen application rates of 300 kg hm^−2^ and 337.5 kg hm^−2^. Hu et al. (2018) [44] studied the effects of nitrogen, phosphorus, and potassium application rates on the yield and quality of the soft rice variety Nanjing 9108 under straw returning conditions. The results showed that, under the condition of full straw returning, fertilizer application rates of N 270 kg hm^−2^, P 108 kg hm^−2^, and K 216 kg hm^−2^ for mechanical transplanted Nanjing 9108 could better coordinate the relationship between a high yield and high quality. Yan et al. (2022) [29] studied the effects of different fertilizer amounts on the yield and quality of Nanjing 46 under the condition of machine-transplanted slow-mixed fertilization. The results showed that with an increase in fertilizer amount, the rice yield showed a trend of first increasing and then decreasing. Applying pure nitrogen 247.5 kg hm^−2^ slow-mixed fertilizer could achieve the highest yield while maintaining rice quality. Zhang (2021) [45] studied the effects of nitrogen fertilizer on the yield and quality of Nanjing 5055 and Nanjing 46 by setting different nitrogen fertilizer application rates and management methods. The results showed that a high yield and high quality could be achieved at a nitrogen fertilizer level of 240–300 kg hm^−2^.

From the average values and significant differences of yield and yield factors in 2021, except for the number of panicles, the yield and yield factors of the N_3_ treatment were not the highest, but there was no significant difference compared to the highest treatment (N_4_). From the average values and significant differences of processing quality and taste quality, although N_3_ was not the treatment with the highest numerical value, there was no significant difference compared to the treatment with the highest numerical value (N_1_ or N_4_). From the average values of RVA characteristics and their significant differences, most traits showed no significant differences between the N_1_ and N_2_ treatments and between the N_4_ and N_3_ treatments, but significant differences between the N_1_ and N_2_ treatments and N_3_ and N_4_ treatments. Therefore, the treatment with a nitrogen fertilizer application of 300 kg hm^−2^ (N_3_) resulted in a yield of 8191 kg hm^−2^ and a taste value of 87.1 points, which could achieve a synergistic improvement in yield and quality. From this, it could be seen that under the conditions of this experiment, N_3_ was the optimal treatment that balanced yield and quality. However, considering the significant differences in most traits between the N_2_ treatment and N_3_ treatment, it was worth further studying whether there was a better treatment level between the N_2_ treatment and N_3_ treatment. So, in 2022, we added two treatments between 150 kg hm^−2^ and 300 kg hm^−2^, setting four treatments of 120 kg hm^−2^ (N_1_), 180 kg hm^−2^ (N_2_), 240 kg hm^−2^ (N_3_), and 300 kg hm^−2^ (N_4_). The results from 2022 showed that the N_4_ treatment had the highest yield, with the highest number of panicles, seed setting rate, and grain weight per plant. However, there was no significant difference in yield, panicle number, and grain weight per plant between the N_4_ treatment and the N_3_ treatment, nor was there a significant difference in the thousand-grain weight between the N_4_ and N_3_ treatments. The N_3_ treatment had the highest taste value, which was significantly different from the N_4_ treatment. The appearance, viscosity, and balance of cooked rice in the N_3_ treatment were also the best, and the protein content was lower. The PFPN was close to 30 kg kg^−1^. The comprehensive results from two years of the experiments indicated that the optimal nitrogen application rate for the variety used in this experiment was 240–300 kg hm^−2^, taking into account both yield and quality. This is consistent with the results of Wei et al. (2012) [22], Hu et al. (2018) [23], Yan et al. (2022) [31], and Zhang (2021) [45]. However, in actual production, the fertilization plan should be flexibly adjusted based on specific variety characteristics, soil fertility conditions, and climatic conditions to achieve the best balance between yield and quality.

It is worth noting that the interaction between the nitrogen application rate and variety for yield factors, processing quality, and rice protein content in 2021 and cooking and taste quality, appearance quality, and peak viscosity in 2022 reached a significance level of 5% or 1%, indicating that there were differences in the responses of different varieties to nitrogen fertilizer for these traits. The Nanjing series semi-glutinous and excellent-tasting *japonica* rice varieties could still maintain a good quality under higher nitrogen application rates, while Nanjingxiangnuo and Nanjing 5758 showed a marked decline in taste quality due to the significant increase in protein content under higher nitrogen application rates, indicating that these two varieties were more sensitive to excessive nitrogen application and suitable nitrogen application rates should be below 240 kg hm^−2^. This indicates that, when formulating fertilization plans, variety characteristics should be taken into account and targeted fertilization should be implemented. In addition, this study only investigated the effect of nitrogen application rate on rice quality. Future research could further explore the comprehensive effects of nitrogen fertilizer management, nitrogen fertilizer types, and other factors on the yield and quality of Nanjing series *japonica* rice, so as to improve high-quality and high-yield cultivation technology systems for Nanjing series *japonica* rice varieties.

## 5. Conclusions

Increasing nitrogen fertilizer application could increase the number of panicles and grains per panicle, ultimately improving yield, while it could also increase the brown rice yield and protein content and reduce PV, FV, and BDV. Applying nitrogen fertilizer could also lead to an increase in the hardness of cooked rice and a decrease in the appearance, viscosity, and balance of cooked rice, as well as a deterioration in gelatinization characteristics, resulting in a significant decrease in taste value. Under the conditions of this experiment, a nitrogen application rate of 240–300 kg hm^−2^ could synergistically improve rice yield and quality.

## Figures and Tables

**Table 1:** Means of yield traits and processing quality for tested varieties at different nitrogen levels in 2021.

N Level	Variety	PN	SP	SSR (%)	TGW (g)	GW (g)	GY (t hm^−2^)	BR (%)	MR (%)	HR (%)
N1	V1	10.5	72.2	93.8	28.7	23.5	5.2	84.4	71.9	70.3
	V2	9.7	100.2	95.3	25.7	23.0	5.9	82.2	67.0	63.4
	V4	12.3	79.5	95.7	25.7	22.5	5.4	81.6	69.0	66.9
	V7	11.4	98.7	92.8	27.8	27.2	5.7	83.1	71.4	66.0
N2	V1	12.0	99.2	95.9	26.1	31.0	6.1	85.7	73.5	71.6
	V2	11.2	91.3	93.1	25.7	22.7	7.4	83.3	66.8	63.4
	V4	12.7	99.7	95.4	25.7	30.4	6.7	83.7	71.9	67.8
	V7	11.3	105.0	95.9	26.1	27.3	7.8	83.9	71.8	69.0
N3	V1	12.7	110.8	88.8	26.2	33.6	7.8	84.7	71.7	68.6
	V2	12.6	108.0	96.6	26.7	27.0	8.8	84.6	70.4	66.1
	V4	13.3	111.9	95.1	24.8	32.1	8.4	85.0	72.3	67.1
	V7	12.2	99.6	95.3	28.7	29.0	7.8	84.3	72.1	68.3
N4	V1	12.2	132.5	86.1	26.7	35.6	7.9	83.9	70.1	64.9
	V2	12.3	93.9	92.6	25.1	26.4	8.9	85.1	73.3	70.6
	V4	11.8	108.3	92.0	25.4	33.7	8.4	85.2	72.9	64.8
	V7	14.1	117.7	94.3	28.7	48.2	8.4	84.7	71.9	66.5
ANOVA	N level (N)	**	**	**	**	**	**	**	NS	NS
	Variety (V)	**	NS	**	**	**	**	**	*	**
	N × V	**	**	**	**	*	NS	**	*	**

*, **: Significant at 5% or 1% levels, respectively, NS: not significant. PN = panicle number per plant, SP = spikelets per panicle, SSR = seed setting rate, TGW = 1000-grain weight, GW = grain weight per plant, GY = grain yield, BR = brown rice percentage, MR = milled rice percentage, and HR = head rice percentage.

**Table 2:** Means of yield traits and processing quality for tested varieties at different nitrogen levels in 2022.

N Level	Variety	PN	SP	SSR (%)	TGW (g)	GW (g)	GY (t hm^−2^)	BR (%)	MR (%)	HR (%)	CG (%)	CD (%)
N1	V2	12.7	75.7	57.2	24.6	16.3	3.3	83.5	69.3	49.3	60.0	12.9
	V3	9.8	89.8	52.6	29.8	14.7	3.0	84.1	68.2	58.9	26.6	7.0
	V5	11.7	90.0	59.2	25.3	16.8	3.3	82.5	65.2	54.4	20.8	4.6
	V6	11.8	91.5	65.8	25.0	22.3	5.4	83.8	68.9	53.0	16.9	2.5
N2	V2	12.7	87.1	64.9	24.2	20.8	5.8	84.9	72.9	65.1	34.0	6.1
	V3	9.9	92.3	69.1	29.2	19.8	6.1	84.7	72.0	67.4	86.4	37.9
	V5	11.1	90.1	65.1	25.0	20.2	6.8	85.4	72.0	66.1	78.8	29.6
	V6	10.4	96.7	82.7	24.2	24.0	6.4	85.5	71.4	62.0	15.0	2.6
N3	V2	12.0	104.2	65.7	23.7	23.3	6.0	84.9	73.4	65.1	40.2	7.0
	V3	9.9	111.8	65.5	28.3	21.7	7.1	85.3	71.9	64.6	73.5	31.7
	V5	11.2	86.6	78.3	24.6	25.0	7.6	86.0	73.0	67.0	56.2	20.2
	V6	12.4	89.4	70.9	24.1	22.9	7.7	85.4	71.7	57.9	17.2	3.4
N4	V2	12.3	107.4	78.4	24.4	29.9	7.7	84.5	73.6	67.8	37.3	6.2
	V3	10.3	93.7	79.4	28.4	24.2	8.4	86.0	73.1	68.6	89.4	39.8
	V5	13.0	92.1	75.1	24.5	25.3	8.2	85.9	73.2	66.8	72.9	29.2
	V6	11.5	76.0	76.6	23.2	23.7	8.1	85.4	71.3	56.6	15.5	3.7
ANOVA	N level (N)	NS	NS	**	**	**	**	**	**	**	**	**
	Variety (V)	**	NS	NS	**	NS	**	NS	NS	**	**	**
	N × V	NS	NS	NS	NS	NS	NS	NS	NS	NS	**	**

**: Significant at 1% levels, NS: not significant. PN = panicle number per plant, SP = spikelets per panicle, SSR = seed setting rate, TGW = 1000-grain weight, GW = grain weight per plant, GY = grain yield, BR = brown rice percentage, MR = milled rice percentage, HR = head rice percentage, CG = chalky grain percentage, and CD = chalkiness degree.

**Table 3:** Means of eating and cooking quality for tested varieties at different nitrogen levels in 2021.

N Level	Variety	AP	HA	ST	BA	TV	PC (%)	PV (cP)	HV (cP)	BDV (cP)	FV (cP)	SBV (cP)	PeT (min)	PaT (°C)
N1	V1	9.5	5.5	9.8	9.7	91.4	3.9	3203	1666	1537	2228	−975	5.8	73.0
	V2	9.7	5.1	9.8	9.8	92.4	4.5	3406	1815	1591	2502	−904	5.6	74.3
	V4	9.2	5.5	9.6	9.4	89.1	4.5	3682	2109	1573	2796	−886	5.8	75.0
	V7	9.8	5.1	9.8	9.8	94.6	4.7	2127	804	1323	999	−1127	3.7	71.0
N2	V1	9.6	5.1	9.8	9.6	91.6	4.1	3103	1554	1548	2091	−1012	5.8	73.6
	V2	9.5	5.3	9.8	9.7	90.8	5.0	3339	1832	1506	2426	−913	5.8	74.9
	V4	8.7	5.7	9.4	8.9	85.9	4.9	3507	1920	1586	2540	−966	5.8	74.1
	V7	9.6	5.5	9.8	9.5	90.3	5.4	2204	885	1319	1100	−1104	3.7	70.9
N3	V1	9.3	5.5	9.7	9.6	90.4	5.6	2956	1688	1268	2195	−761	6.0	74.2
	V2	8.7	5.9	9.4	8.9	86.0	5.6	3140	1724	1415	2281	−859	5.8	74.2
	V4	8.4	6.2	9.3	8.6	83.7	6.0	3355	1917	1438	2475	−880	6.0	74.1
	V7	9.3	5.7	9.7	9.2	88.3	6.3	2095	750	1345	929	−1166	3.7	71.5
N4	V1	8.2	6.5	9.0	8.3	82.1	6.5	2977	1636	1341	2150	−827	6.0	73.7
	V2	8.9	5.8	9.6	9.1	87.2	5.8	3161	1726	1436	2297	−865	5.8	74.1
	V4	8.3	6.3	9.1	8.5	82.9	5.9	3258	1799	1459	2342	−916	6.0	74.4
	V7	8.8	6.1	9.4	8.8	85.0	7.1	2023	713	1310	890	−1133	3.7	71.8
ANOVA	N level (N)	**	**	**	**	**	**	**	NS	**	**	NS	**	NS
	Variety (V)	**	NS	*	**	**	**	**	**	**	**	**	**	**
	N × V	NS	NS	NS	NS	NS	**	NS	NS	NS	NS	NS	NS	NS

*, **: Significant at 5% or 1% levels, respectively, NS: not significant. AP = appearance of cooked rice, HA = hardness of cooked rice, ST = stickiness of cooked rice, BA = balance of cooked rice, TV = taste value, PC = protein content, PV = peak viscosity, HV = hot viscosity, BDV = breakdown viscosity, FV = final viscosity, SBV = setback viscosity, PeT = peak time, and PaT = pasting temperature.

**Table 4:** Means of eating and cooking quality for tested varieties at different nitrogen levels in 2022.

N Level	Variety	AC (%)	GC (mm)	AP	HA	ST	BA	TV	PC (%)	PV (cP)	HV (cP)	BDV (cP)	FV (cP)	SBV (cP)	PeT (min)	PaT (°C)
N1	V2	9.5	85.3	8.4	5.9	8.8	8.4	82.0	6.5	2786	1630	1156	2304	−482	6.1	72.5
	V3	11.3	85.3	6.4	6.9	6.3	6.0	66.5	7.4	3395	1749	1645	2167	−1227	6.0	69.6
	V5	11.6	75.0	8.2	6.1	7.7	7.7	77.4	6.5	2630	1526	1104	2141	−489	6.1	73.3
	V6	19.4	80.7	8.6	5.9	9.0	8.6	83.8	5.8	3184	2165	1019	2963	−221	6.5	72.0
N2	V2	9.0	79.3	8.9	5.6	9.4	9.0	86.3	7.4	2502	1380	1123	2002	−500	6.0	72.2
	V3	11.3	79.0	6.4	6.8	6.5	6.2	67.8	7.8	2964	1594	1371	2135	−829	6.1	73.2
	V5	13.0	92.0	8.1	5.9	7.9	7.9	78.9	7.3	2972	1717	1255	2357	−615	6.2	73.6
	V6	19.1	71.7	7.3	6.4	8.1	7.5	76.0	8.1	2823	2091	731.3	3087	264.7	6.8	71.7
N3	V2	9.6	51.0	9.0	5.5	9.4	9.1	87.4	6.5	2501	1557	943.7	2262	−239	6.2	69.6
	V3	11.3	89.7	7.2	6.5	7.3	7.0	72.9	7.5	3287	1886	1400	2456	−830	6.2	72.7
	V5	13.2	83.7	8.6	5.7	8.5	8.5	82.3	6.6	3067	1740	1326	2393	−674	6.1	73.1
	V6	19.0	74.0	7.9	6.1	8.5	8.0	79.5	6.2	2843	2053	790.3	3049	205.7	6.7	71.5
N4	V2	9.4	71.0	8.7	5.7	9.2	8.8	85.1	6.6	2841	1562	1278	2191	−650	6.0	70.6
	V3	11.0	79.7	6.7	6.6	6.9	6.6	69.9	7.6	3255	1820	1435	2394	−861	6.1	72.5
	V5	11.4	84.0	8.2	6.0	8.0	8.0	79.1	7.6	2847	1714	1133	2361	−487	6.2	73.0
	V6	18.7	72.3	6.2	6.9	7.2	6.4	68.8	7.1	2628	1827	801	2921	292.3	6.6	70.9
ANOVA	N level (N)	NS	**	**	**	*	**	**	**	NS	NS	NS	NS	NS	NS	NS
	Variety (V)	**	**	**	**	**	**	**	**	**	**	**	**	**	**	NS
	N × V	NS	**	**	**	**	**	**	**	*	NS	NS	NS	NS	NS	NS

*, **: Significant at 5% or 1% levels, respectively, NS: not significant. AC = amylose content, GC = gel consistency, AP = appearance of cooked rice, HA = hardness of cooked rice, ST = stickiness of cooked rice, BA = balance of cooked rice, TV = taste value, PC = protein content, PV = peak viscosity, HV = hot viscosity, BDV = breakdown viscosity, FV = final viscosity, SBV = setback viscosity, PeT = peak time, and PaT = pasting temperature.

**Table 5:** Means of yield traits and processing quality for tested traits at different nitrogen levels in 2021.

N Level	PN	SP	SSR (%)	TGW (g)	GW (g)	GY (t hm^−2^)	BR (%)	MR (%)	HR (%)	ANUE (kg kg^−1^)	PFPN (kg kg^−1^)
N1	11.0 ± 1.2	87.7 ± 13.9	94.4 ± 1.3	27.0 ± 1.5	24.1 ± 2.2	5.6 ± 0.3	82.8 ± 1.2	69.8 ± 2.3	66.6 ± 2.9	—	—
N2	11.8 ± 0.7	98.8 ± 5.7	95.1 ± 1.3	25.9 ± 0.3	27.8 ± 3.8	7.0 ± 0.7	84.2 ± 1.1	71.0 ± 2.9	68.0 ± 3.4	9.5 ± 3.2	46.7 ± 4.9
N3	12.7 ± 0.5	107.6 ± 5.6	93.9 ± 3.5	26.6 ± 1.6	30.4 ± 3.0	8.2 ± 0.5	84.6 ± 0.3	71.6 ± 0.9	67.5 ± 1.2	8.7 ± 1.3	27.3 ± 1.6
N4	12.6 ± 1.0	113.1 ± 16.2	91.3 ± 3.6	26.5 ± 1.7	36.0 ± 9.1	8.4 ± 0.4	84.8 ± 0.6	72.0 ± 1.4	66.7 ± 2.7	6.3 ± 0.4	18.7 ± 0.9

PN = panicle number per plant, SP = spikelets per panicle, SSR = seed setting rate, TGW = 1000-grain weight, GW = grain weight per plant, GY = grain yield, BR = brown rice percentage, MR = milled rice percentage, HR = head rice percentage, ANUE = agronomic nitrogen use efficiency, and PFPN = partial factor productivity of nitrogen.

**Table 6:** Means of yield traits, processing, and appearance quality for tested traits at different nitrogen levels in 2022.

N Level	PN	SP	SSR (%)	TGW (g)	GW (g)	GY (t hm^−2^)	BR (%)	MR (%)	HR (%)	CG (%)	CD (%)	PFPN (kg kg^−1^)
N1	11.5 ± 1.2	86.7 ± 7.4	58.7 ± 5.5	26.1 ± 2.4	17.5 ± 3.3	3.7 ± 0.3	83.5 ± 0.7	67.9 ± 1.8	53.9 ± 4.0	31.1 ± 19.7	6.8 ± 4.5	31.1 ± 9.3
N2	11.1 ± 1.2	91.5 ± 4.1	70.5 ± 8.4	25.6 ± 2.4	21.2 ± 1.9	6.3 ± 0.7	85.1 ± 0.4	72.1 ± 0.6	65.2 ± 2.3	53.6 ± 34.6	19.0 ± 17.4	34.8 ± 2.3
N3	11.4 ± 1.1	98.0 ± 12.0	70.1 ± 6.0	25.2 ± 2.1	23.2 ± 1.3	7.1 ± 0.5	85.4 ± 0.4	72.5 ± 0.8	63.7 ± 4.0	46.8 ± 23.9	15.6 ± 13.0	29.6 ± 3.2
N4	11.8 ± 1.1	92.3 ± 12.9	77.3 ± 1.9	25.1 ± 2.3	25.7 ± 2.8	8.1 ± 0.4	85.4 ± 0.7	72.8 ± 1.0	65.0 ± 5.6	53.8 ± 33.5	19.7 ± 17.6	27.0 ± 1.0

PN = panicle number per plant, SP = spikelets per panicle, SSR = seed setting rate, TGW = 1000-grain weight, GW = grain weight per plant, GY = grain yield, BR = brown rice percentage, MR = milled rice percentage, HR = head rice percentage, CG = chalky grain percentage, CD = chalkiness degree, and PFPN = partial factor productivity of nitrogen.

**Table 7:** Means of eating and cooking quality for tested traits at different nitrogen levels in 2021.

N Level	AP	HA	ST	BA	TV	PC (%)	PV (cP)	HV (cP)	BDV (cP)	FV (cP)	SBV (cP)	PeT (min)	PaT (°C)
N1	9.5 ± 0.3	5.3 ± 0.2	9.8 ± 0.1	9.7 ± 0.2	91.9 ± 2.3	4.4 ± 0.3	3104 ± 681	1598 ± 561	1506 ± 124	2131 ± 789	−973 ± 110	5.2 ± 1.0	73.3 ± 1.8
N2	9.3 ± 0.4	5.4 ± 0.3	9.7 ± 0.2	9.4 ± 0.4	89.6 ± 2.5	4.8 ± 0.5	3038 ± 580	1548 ± 469	1490 ± 118	2039 ± 655	−999 ± 81	5.3 ± 1.1	73.4 ± 1.7
N3	8.9 ± 0.5	5.8 ± 0.3	9.5 ± 0.2	9.1 ± 0.4	87.1 ± 2.9	5.9 ± 0.3	2886 ± 552	1520 ± 523	1367 ± 77	1970 ± 704	−916 ± 174	5.4 ± 1.1	73.5 ± 1.3
N4	8.5 ± 0.4	6.2 ± 0.3	9.3 ± 0.3	8.7 ± 0.3	84.3 ± 2.3	6.3 ± 0.6	2855 ± 567	1468 ± 508	1387 ± 72	1920 ± 691	−935 ± 137	5.4 ± 1.1	73.5 ± 1.2

AP = appearance of cooked rice, HA = hardness of cooked rice, ST = stickiness of cooked rice, BA = balance of cooked rice, TV = taste value, PC = protein content, PV = peak viscosity, HV = hot viscosity, BDV = breakdown viscosity, FV = final viscosity, SBV = setback viscosity, PeT = peak time, and PaT = pasting temperature.

**Table 8:** Means of eating and cooking quality for tested traits at different nitrogen levels in 2022.

N Level	AC (%)	GC (mm)	AP	HA	ST	BA	TV	PC (%)	PV (cP)	HV (cP)	BDV (cP)	FV (cP)	SBV (cP)	PeT (min)	PaT (°C)
N1	13.0 ± 4.4	81.6 ± 4.9	7.9 ± 1.0	6.2 ± 0.5	8.0 ± 1.2	7.7 ± 1.2	77.4 ± 7.8	6.6 ± 0.7	2999 ± 352	1768 ± 281	1231 ± 282	2394 ± 386	-605 ± 433	6.2 ± 0.3	71.9 ± 1.6
N2	13.1 ± 4.3	80.5 ± 8.4	7.7 ± 1.1	6.2 ± 0.5	8.0 ± 1.2	7.7 ± 1.1	77.2 ± 7.7	7.6 ± 0.3	2815 ± 220	1695 ± 298	1120 ± 278	2395 ± 484	-420 ± 476	6.3 ± 0.3	72.7 ± 0.9
N3	13.3 ± 4.1	74.6 ± 17.0	8.2 ± 0.8	6.0 ± 0.4	8.5 ± 0.9	8.2 ± 0.9	80.5 ± 6.0	6.7 ± 0.6	2924 ± 335	1809 ± 211	1115 ± 295	2540 ± 349	-384 ± 466	6.3 ± 0.3	71.7 ± 1.6
N4	12.6 ± 4.2	76.8 ± 6.2	7.4 ± 1.2	6.3 ± 0.6	7.8 ± 1.0	7.4 ± 1.1	75.7 ± 7.8	7.2 ± 0.5	2893 ± 262	1731 ± 124	1162 ± 270	2467 ± 316	-426 ± 503	6.2 ± 0.2	71.8 ± 1.2

AC = amylose content, GC = gel consistency, AP = appearance of cooked rice, HA = hardness of cooked rice, ST = stickiness of cooked rice, BA = balance of cooked rice, TV = taste value, PC = protein content, PV = peak viscosity, HV = hot viscosity, BDV = breakdown viscosity, FV = final viscosity, SBV = setback viscosity, PeT = peak time, and PaT = pasting temperature.

**Table 9:** Correlation coefficients between different traits.

Character	PN	SP	SSR (%)	TGW (g)	GW (g)	GY (t hm^−2^)	BR (%)	MR (%)	HR (%)	AP	HA	ST	BA	TV	PC (%)	PV (cP)	HV (cP)	BDV (cP)	FV (cP)	SBV (cP)	PeT (min)	PaT (°C)
PN		−0.264	−0.002	−0.772 **	0.242	0.060	−0.144	0.115	−0.237	0.727 **	−0.713 **	0.767 **	0.762 **	0.760 **	−0.526 *	−0.653 **	−0.269	−0.470	−0.027	0.395	0.043	−0.006
SP	0.470		0.173	0.243	0.415	0.242	0.141	0.334	0.538 *	0.140	−0.170	0.095	0.124	0.135	0.144	0.219	0.040	0.200	−0.124	−0.241	−0.078	−0.185
SSR (%)	−0.039	−0.471		−0.306	0.866 **	0.838 **	0.703 **	0.643 **	0.510 *	−0.079	0.014	0.085	0.027	0.014	0.240	−0.072	0.303	−0.335	0.431	0.401	0.427	0.086
TGW (g)	0.018	−0.077	0.014		−0.413	−0.221	−0.059	−0.166	0.223	−0.622 *	0.618 *	−0.764 **	−0.711 **	−0.699 **	0.441	0.746 **	−0.001	0.800 **	−0.354	−0.767 **	−0.449	0.078
GW (g)	0.722 **	0.720 **	−0.298	0.268		0.846 **	0.598 *	0.738 **	0.548 *	0.229	−0.294	0.373	0.327	0.325	−0.032	−0.122	0.173	−0.278	0.286	0.313	0.285	−0.101
GY (t/hm^2^)	0.565 *	0.641 **	−0.204	−0.216	0.484		0.850 **	0.800 **	0.622 *	−0.082	−0.009	0.039	0.007	0.001	0.192	0.014	0.246	−0.194	0.336	0.268	0.329	0.112
BR (%)	0.426	0.330	−0.118	0.031	0.500 *	0.576 *		0.819 **	0.664 **	−0.198	0.105	−0.092	−0.120	−0.127	0.448	0.154	0.274	−0.067	0.296	0.144	0.286	0.054
MR (%)	0.466	0.135	0.012	0.131	0.434	0.300	0.782 **		0.779 **	0.123	−0.233	0.246	0.216	0.224	0.292	−0.089	−0.086	−0.022	−0.016	0.044	0.041	−0.062
HR (%)	0.176	−0.310	0.198	0.145	−0.001	−0.079	0.511 *	0.737 **		−0.022	−0.079	−0.098	−0.037	−0.025	0.529 *	0.115	−0.273	0.355	−0.349	−0.360	−0.295	0.100
AP	−0.599 *	−0.564 *	0.329	0.269	−0.505 *	−0.631 **	−0.363	−0.266	0.200		−0.990 **	0.915 **	0.979 **	0.977 **	−0.565 *	−0.440	−0.276	−0.237	−0.189	0.126	−0.127	0.076
HA	0.591 *	0.606 *	−0.365	−0.069	0.586 *	0.691 **	0.383	0.289	−0.178	−0.953 **		−0.910 **	−0.975 **	−0.976 **	0.518 *	0.439	0.298	0.217	0.207	−0.110	0.138	−0.085
ST	−0.533 *	−0.674 **	0.397	0.193	−0.559 *	−0.562*	−0.306	−0.229	0.291	0.967 **	−0.929 **		0.977 **	0.977 **	−0.568 *	−0.542 *	−0.123	−0.476	0.059	0.395	0.138	−0.129
BA	−0.622 *	−0.630 **	0.278	0.169	−0.583 *	−0.648 **	−0.367	−0.305	0.198	0.979 **	−0.966 **	0.972 **		0.999 **	−0.567 *	−0.500 *	−0.202	−0.364	−0.064	0.267	0.010	−0.007
TV	−0.616 *	−0.598 *	0.265	0.212	−0.557 *	−0.690 **	−0.379	−0.266	0.185	0.979 **	−0.972 **	0.947 **	0.986 **		−0.567 *	−0.49 8*	−0.218	−0.348	−0.083	0.250	−0.008	−0.032
PC (%)	0.658 **	0.748 **	−0.345	0.127	0.726 **	0.839 **	0.367	0.232	−0.211	−0.645 **	0.778 **	−0.641 **	−0.718 **	−0.724 **		0.171	−0.098	0.267	−0.171	−0.250	−0.098	0.184
PV (cP)	−0.188	−0.313	0.025	−0.679 **	−0.444	−0.200	−0.185	−0.310	−0.132	−0.252	0.026	−0.157	−0.095	−0.151	−0.445		0.478	0.665 **	0.079	−0.574 *	−0.141	0.090
HV (cP)	−0.125	−0.228	−0.076	−0.708 **	−0.396	−0.108	−0.143	−0.290	−0.146	−0.319	0.100	−0.221	−0.151	−0.212	−0.362	0.989 **		−0.338	0.901 **	0.435	0.786 **	−0.042
BDV (cP)	−0.401	−0.588 *	0.443	−0.373	−0.529 *	−0.532 *	−0.313	−0.307	−0.036	0.101	−0.291	0.154	0.170	0.143	−0.676 **	0.777 **	0.674 **		−0.681 **	−0.984 **	−0.819 **	0.131
FV (cP)	−0.160	−0.244	−0.063	−0.696 **	−0.412	−0.135	−0.157	−0.310	−0.145	−0.288	0.069	−0.194	−0.122	−0.183	−0.387	0.993 **	0.998 **	0.700 **		0.771 **	0.957 **	−0.109
SBV (cP)	0.000	0.115	−0.445	−0.634 **	−0.182	0.183	−0.001	−0.253	−0.176	−0.395	0.249	−0.320	−0.224	−0.287	−0.049	0.761 **	0.842 **	0.214	0.835 **		0.876 **	−0.147
PeT (min)	−0.071	−0.079	−0.255	−0.629 **	−0.245	0.001	0.101	−0.144	−0.055	−0.392	0.189	−0.310	−0.223	−0.279	−0.267	0.917 **	0.948 **	0.539 *	0.947 **	0.892 **		−0.120
PaT (°C)	−0.029	−0.127	−0.125	−0.683 **	−0.272	0.026	−0.098	−0.359	−0.255	−0.348	0.138	−0.252	−0.186	−0.259	−0.209	0.932 **	0.952 **	0.599 *	0.952 **	0.852 **	0.923 **	

The lower left and upper right corners of the diagonal in the table represent the correlation coefficients for 2021 and 2022, respectively. PN = panicle number per plant, SP = spikelets per panicle, SSR = seed setting rate, TGW = 1000-grain weight, GW = grain weight per plant, GY = grain yield, BR = brown rice percentage, MR = milled rice percentage, HR = head rice percentage, AP = appearance of cooked rice, HA = hardness of cooked rice, ST = stickiness of cooked rice, BA = balance of cooked rice, TV = taste value, PC = protein content, PV = peak viscosity, HV = hot viscosity, BDV = breakdown viscosity, FV = final viscosity, SBV = setback viscosity, PeT = peak time, PaT = pasting temperature, CG = chalky grain percentage, CD = chalkiness degree, AC = amylose content, GC = gel consistency, and PFPN = partial factor productivity of nitrogen. *, **: Significant at 5% or 1% levels, respectively.

## Data Availability

The datasets presented in this study are included in the main text; further inquiries can be directed to the corresponding author.

## Abbreviations

AC	Amylose content
ANOVA	Analysis of variance
ANUE	Agronomic nitrogen use efficiency
BDV	Breakdown viscosity
cP	Centipoise
CSV	Consistency viscosity
FV	Final viscosity
GC	Gel consistency
GT	Gelatinization temperature
HV	Hot viscosity
MS	Mean square
PaT	Pasting temperature
PC	Protein content
PeT	Peak time
PFPN	Partial factor productivity of nitrogen
PV	Peak viscosity
RVA	Rapid Visco-analyzer
SBV	Setback viscosity
TV	Taste value
